# Intracranial Hemorrhage—Is Very Early Rehabilitation Safe? A Narrative Review

**DOI:** 10.3390/jcm13133776

**Published:** 2024-06-27

**Authors:** Klaudia Marek, Ewa Zielińska-Nowak, Justyna Redlicka, Michał Starosta, Elżbieta Miller

**Affiliations:** Department of Neurological Rehabilitation, Medical University of Lodz, Milionowa 14, 93-113 Lodz, Poland; ewa.zielinska@umed.lodz.pl (E.Z.-N.); justyna.redlicka@umed.lodz.pl (J.R.); michal.starosta@umed.lodz.pl (M.S.)

**Keywords:** intracerebral hemorrhage, stroke, early rehabilitation

## Abstract

Intracerebral hemorrhage (ICH) is a serious neurological disease with a 30-day mortality rate of 34–50%. Rehabilitation can reduce disability and improve recovery from a stroke; however, it is uncertain whether early rehabilitation is safe. There are many studies and reviews on rehabilitation for chronic conditions, but there is not enough information on the details of rehabilitation in the acute and subacute phases of ICH. We analyzed clinical trials from the electronic databases PubMed, PubMedCentral, Medline, Cochrane Library, Embase, Scopus and PEDro. Based on the data, we determined that early rehabilitation of patients with ICH has beneficial effects on improving ADL scores, motor function, functional independence, quality of life, improved gait, improved trunk control and reduced mortality. Varying the duration and intensity of rehabilitation in patients with ICH may improve health status, functional outcomes and reduce the length of stay in the hospital. The earliest protocol for initiating rehabilitation after ICH included up to 24 h after stroke onset. The medical literature indicates the need for more randomized controlled group trials of early rehabilitation in patients with acute and subacute ICH with a precise timing of rehabilitation initiation. This narrative review aims to summarize the existing evidence and provide insights into the current state of knowledge regarding the safety of early rehabilitation. There is a need for a clear definition of “early rehabilitation” when determining the most appropriate time to begin rehabilitation therapy.

## 1. Introduction

Intracranial hemorrhage (ICH) is a critical and often life-threatening condition characterized by bleeding within the intracranial vault, including the brain parenchyma and surrounding meningeal spaces. Intracranial hemorrhage is defined as any bleeding within the intracranial vault [1]. The mortality rate is extremely high, reaching approximately 50% within the first month and for those who survive, there is a notable risk of severe disability [2]. Although researchers initially focused on decreasing mortality rates, recent endeavors have shifted towards addressing disability among survivors. The paradigm of care for patients with ICH has evolved, reflecting a growing emphasis on a comprehensive, multidisciplinary approach to optimize outcomes [3].

**Figure 1:**
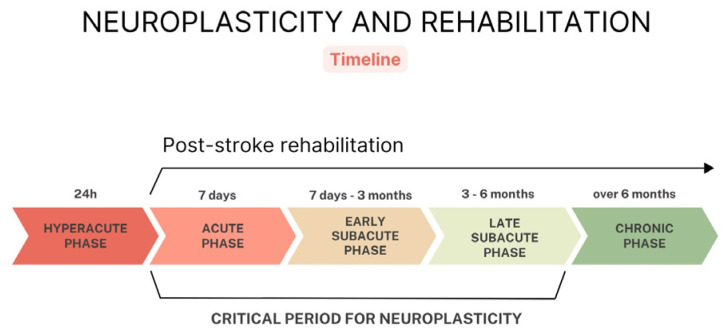
Neuroplasticity and rehabilitation.

Research suggests that early physical rehabilitation for ICH survivors may result in improved motor recovery, reduced functional and neurological impairment, as well as an enhanced quality of life [4,5]. In addition to its impact on physical functioning, the influence of early rehabilitation on the mental health of individuals with ICH has been investigated. Patients engaged in early rehabilitation protocols often exhibit lower levels of depression and anxiety compared to those receiving standard care [5]. In general, recovery is quicker in the first few weeks after an ICH, but can take several months. Approximately half of the survivors may still need help with daily activities. The preparation of the patient’s family is therefore very important. In order for rehabilitation to be optimal and correct, caregivers and families need to be shown how exercises should be carried out at home. Knowledge and involvement from the family can have a positive impact on home rehabilitation as well as on the patient’s mental state [6]. Patients after ICH need comprehensive rehabilitation, considering cognitive functions, mental disorders, spasticity and functional scales. A significant role of early rehabilitation was observed among patients in poor clinical conditions [7]. While planning rehabilitation, it is also worth taking into account that neuroplasticity is most active during the acute (24 h–7 days) and subacute phases (7 days–6 months); hence, the initiation of rehabilitation should be tailored to take advantage of the brain’s ability to adapt and reorganize, which can accelerate functional recovery and optimize outcomes in stroke survivors (Figure 1) [8].

Despite promising results, there is an ongoing debate among specialists about the optimal moment to start rehabilitation. According to guidelines, patients should be closely monitored in the initial period after ICH, especially with regard to blood pressure, which also leads to caution in starting rehabilitation [9].

Recovery from stroke, including hemorrhagic stroke, is heterogeneous in terms of patient outcomes [10]. A study by Oki et al., 2024 provided implications for clinical practice based on baseline data from health facilities. Certain characteristics of specific health facilities influence the early initiation of active physical activity and the early mobilization of patients, resulting in better achievement of health status indicators [11]. Early acute rehabilitation in post-stroke patients has a beneficial effect on the respiratory, immune, skeletal and, importantly, cardiovascular systems, whose dysfunction may have influenced the previous development of stroke [12]. Exercise reduces the risk of serious complications such as deep vein thrombosis or phlebitis. Mobilization, positioning therapy and health education show benefits in post-stroke patients with shoulder pain, which is not uncommon [13]. In the first 24 h after stroke, Skarin et al., 2011 investigated the prevalence of various staff concerns about early mobilization away from the patient’s bedside. The study was conducted among professionals caring for stroke patients. Up to 60% of staff had concerns about early mobilization, with significantly more professionals concerned about early mobilization in patients with ICH (59%) than in patients with ischemic stroke (23%) [14].

Studies show that the provision of early mobilization for stroke patients is a differentiating factor between care in a stroke unit and care in a general ward or other specification. The provision of specialist neurological care, through the presence of specialists who deal exclusively with stroke and its complications, is associated with better patient outcomes [15]. Regardless of the type of stroke, in the acute phase, treatment and rehabilitation in a stroke unit increase patients’ chances of survival and functional independence [16]. Outcomes for patients after ischemic and hemorrhagic strokes are inconclusive and mixed. There are some studies that show a better recovery for patients after ICH, but there are also some that suggest the opposite, that patients after ischemic stroke have a better outcome [17,18,19]. However, the study by Oosterveer et al., 2022 provides us with evidence that the outcomes of patients in rehabilitation after 3–6 months do not differ significantly between ischemic and hemorrhagic strokes, regardless of the type of hospital treatment they received [20].

Available information from the literature suggests that the majority of people who have had a hemorrhagic stroke recover their motor function within the first 3–6 months after the event [21]. The results obtained by Salvadori et al., 2020 showed that patients with ICH had a worse functional and clinical status on admission to the intensive rehabilitation unit compared with those with ischemic stroke. Patients with ICH were younger, required a longer and more intensive hospital stay and had more initial complications compared to patients with ischemic stroke [22]. The timing of early rehabilitation after a stroke is unclear. According to the AHA 2022 guidelines for hemorrhagic stroke, it seems most appropriate to start rehabilitation within 24–48 h, but not before the end of 24 h after stroke onset [6]. Patients mobilized within 24 h of stroke onset showed detrimental effects at 3 months, according to the mRS scale [23]. Findings also suggest that early mobilization may increase the risk of cerebral rebleeding and affect poststroke hypertension [24]. However, these concerns and findings raise questions [25]. Few studies of early rehabilitation have included patients only after ICH and often did not stratify stroke subtypes for randomization and did not determine expected effects [26].

There is a need for a clear definition of “early rehabilitation” when determining the most suitable time to start rehabilitation therapy, as these timeframes often vary significantly between studies [27]. Integrating rehabilitation into the acute management of ICH poses great challenges and requires an understanding of the underlying pathophysiology and patient characteristics. It is important for healthcare providers to carefully assess the risks and benefits of early rehabilitation for each individual patient after ICH.

This narrative review aims to summarize the existing evidence and provide insights into the current state of knowledge regarding the safety of early rehabilitation interventions in the acute and subacute phases of ICH care. We will try to compare the risks and benefits of early rehabilitation and identify potential future directions for research.

## 2. Materials and Methods

Electronic databases PubMed, PubMedCentral, Medline, Cochrane Library, Embase, Scopus, PEDro were comprehensively searched. This systematic review was conducted manually. The PEDro search engine was used at the very end of the study as a quality assessment tool to check the methodological quality of the specific studies that qualified for review.

Keywords and keyword combinations were used:

- Early rehabilitation of intracerebral hemorrhage (ICH);
- Acute rehabilitation of intracerebral hemorrhage (ICH);
- Rehabilitation of intracerebral hemorrhage (ICH);
- Mortality of rehabilitation intracerebral hemorrhage (ICH);

The study was checked as below, and the following data were expected:

- Type of study conducted;
- Characteristics of patients (condition, type of hemorrhage/stroke);
- Number of patients participating in the study;
- The time since the onset of the stroke;
- Duration of the study/rehabilitation;
- Type of rehabilitation;
- Mortality rate during the study;
- Outcome measures used and conclusions.

If three or more data points were missing, the study was excluded due to the insufficient information presented. Study participants were patients with ICH within a short time of stroke onset.

**Figure 2:**
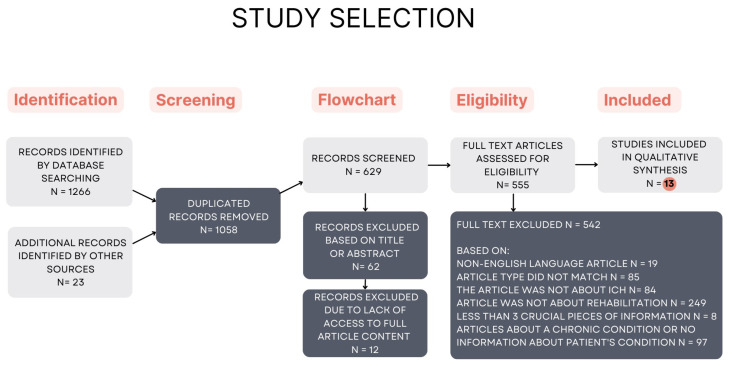
Flowchart of study selection.

Qualified articles were published only in English with open access. Articles available only with an abstract, in a language other than English, on the rehabilitation of patients with ICH in a chronic condition or with an undetermined condition (no time given when the stroke occurred), on patients with subarachnoid hemorrhage, were eliminated and not included in the review. For this reason, only 13 studies meeting all requirements were accepted and included in our quality synthesis (Figure 2). This systematic review aims to answer the research question of whether early rehabilitation for patients with ICH is safe as well as effective.

## 3. Results

Rehabilitation is an important part of treatment, and has been used successfully for several decades, leading to improved functional conditions after stroke with ICH [28,29]. There is a noticeable increase in research after 2019 on rehabilitation and treatment of patients in the acute phase after ICH [4,5,18,25,27,28,30,31,32,33,34,35,36]. A systematic review discussing the efficacy and safety of early rehabilitation is missing from recent publications. A total of 13 articles were analyzed. In the systematic review, we allowed the inclusion of articles mainly from the last 12 years. Details of the studies included in the systematic review are discussed in Table 1.

The number of patients in the results obtained varied from 40 to as many as 85,664 people. The numbers expressed in thousands of subjects obtained in the review come from multicenter studies whose authors collected many details of improvements in patient indicators, rehabilitation and length of stay in the ward, as well as checking the ward specifications. In the study by Sun et al. 2018, the researchers collected written care protocols, thus monitoring the results achieved and their evidence-based measures [37]. The duration of a single rehabilitation session ranged from 30 min to 3 h. The most commonly used outcome measures were the National Institutes of Health Stroke Scale, reported in six studies, and the Modified Rankin Scale, reported in five studies. In-hospital mortality of ICH patients was reported in nine studies, while four studies did not address this issue and did not report a measure. Of the 13 studies included in the review, 5 received a PEDro database score ranging from 0 to 8, with one article receiving a score of 5, one article receiving a score of 7, three articles receiving a score of 8 and eight articles receiving a score of 0.

**Figure 3:**
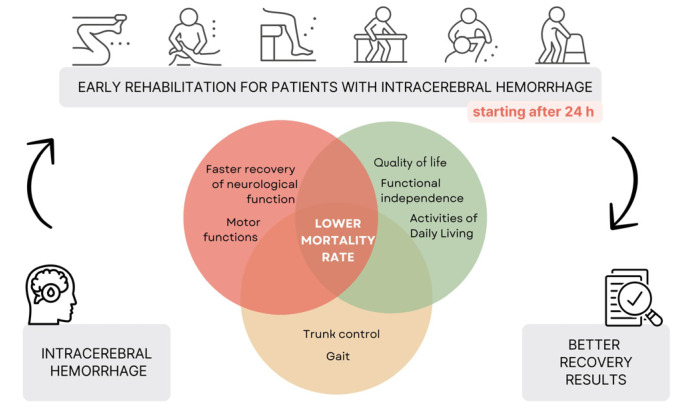
Summary of review results.

A transparent summary of the results of the early rehabilitation of patients with ICH is presented in Figure 2. Appropriate early rehabilitation in patients after ICH has many potential benefits related to functional status, which has an impact on reducing hospitalization in the ward and mortality. Each of the articles selected for review outlines the benefits of starting rehabilitation (Figure 3).

The total number of records retrieved, consisting of records identified by searching medical databases and additional records identified by other sources (detailed manual searches), was 1277 (Table 2). Details of the searches for records that were later qualified for systematic review in specific databases are shown in Table 3. Searches for records in databases were possible using the following settings: Abstract, Title and Keywords.

## 4. Discussion

The purpose of our study was to perform a systematic review of early rehabilitation for patients with ICH. Verifying the safety of the use of early rehabilitation is crucial due to the high mortality rate occurring in patients with ICH, reaching up to 50% within 1 month [2]. There is growing evidence in the scientific world of the effectiveness of multidisciplinary neurological rehabilitation for post-stroke patients [38]. Unfortunately, a larger number of rehabilitation studies focus on ischemic stroke. The basis of therapy for patients with ICH is based on the general principles used in the rehabilitation of patients after an ischemic stroke [3].

We analyzed a total of 13 scientific articles comparing the number of patients, specificity and type of stroke, time since stroke, rehabilitation and treatment time, type of rehabilitation used, mortality, outcome measures and conclusions. Only studies involving patients with ICH who had recently experienced a stroke were eligible for synthesis. Studies involving patients in the chronic phase were excluded. The studies included in the analysis were performed in the following countries: China, Australia, Japan, Taiwan, Germany, USA. As many as nine studies were conducted exclusively on patients with a history of ICH [4,5,25,27,28,30,32,33,34], four studies included patients with ICH and ischemic stroke [31,35,36], and one study included patients with ICH and cerebral infarction (CI) [18].

Acute conditions affected patients in as many as 10 studies [4,25,28,30,31,32,33,34,35,36], subacute conditions in 1 [5]. In one study, clinicians classified patients into acute, subacute and chronic conditions [18]. The one study included in the review did not provide detailed information on patient status and time since stroke onset; however, this study contained most of the other valuable information that made the systematic review more interesting, so it was not excluded at this time [27]. In seven studies, we noted detailed data on the time from stroke onset to admission to the ward and the start of treatment and rehabilitation. This included a time range of 24 h [35,36], 48 h [4,25,32,33] and 72 h [31]. Providing information on the duration of the patient’s stay during the examinations conducted or the duration of the examination/rehabilitation was problematic in four articles [4,27,31,33,34]. The duration of rehabilitation and hospitalization for patients varied. Long rehabilitations lasted 6 months or 3 months [5,25]. Short ones lasted 9 days [31], approximately 14 days [28,35,36], more than 15 days [30]. One study had a duration of 8 weeks [18]. Many studies confirm that dedicated stroke units provide better patient outcomes and rehabilitation compared to general units [39]. Attention should be paid to controlling the patient’s blood pressure and medical condition during rehabilitation [32].

In our systematic review, patients were hospitalized and received rehabilitation in the following departments: emergency or neurology department [5], stroke center [25], comprehensive inpatient rehabilitation [18,30], acute hospitalization [30], intensive care [30], emergency [30] and stroke [4,30,35]. Many researchers did not specify the ward [27,28,31,32,33,34,36]. The description of the duration of rehabilitation is often lacking in detail [27,28,30,32,33,34,36]. The shortest rehabilitation time was 30 min per day [5,25]. Most often, patients received rehabilitation 5 days a week without weekends [5,25]. Patients were also treated 7 days a week for 2–3 h [18] or 40 min per day [31]. Most rehabilitation programs for patients with ICH have included standard therapies, including occupational therapy, physical therapy, kinesitherapy, task training, ADL training and gait training [4,5,18,27,28,30,33]. Specific therapies such as HAL exoskeleton rehabilitation [34], mobilization within 24 h of ICH [35,36], early mobilization within 24–72 h of stroke onset along with standard early rehabilitation, Vojta neurophysiological method [31] and acupuncture along with standard rehabilitation [32] have been performed. Early initiation of rehabilitation in patients with ICH can lead to improvements in ADLs [5,32], motor function [4,5,28,32,36], quality of life [28,32], functional independence [5,25,36], better postural control [31], accelerated recovery of neurological function [28,32] and has been shown to reduce mortality among post-stroke patients [30]. The number of post-stroke patients included in the trials ranged from 40 to 85,664, with no information on mortality found in four trials [27,28,32,33]. The number of deaths with a high percentage of mortality occurred in the study by Ogata et al., (2015) [34], with 156 deaths, representing 57.7% of all patients participating in the study. However, in the vast majority of cases, mortality was exceptionally low or absent [4,5,18,25,31,35,36].

PEDro was used to evaluate the original articles included in the synthesis and listed in Table 1. The scale includes internal validity and interpretability to assess the methodological quality of the study performed. The lowest score is 0, and the highest score is 10. As many as eight studies received a score of 0 on the PEDro scale [18,25,27,28,30,32,33,34]. The scores obtained were in the range: 5 [5], 7 [32] and 8 [4,35,36]. The type of rehabilitation that appeared twice in the review was the AVERT protocol. This involves starting to mobilize the patient as early as possible, within 24 h of the onset of stroke symptoms. The researchers who used the AVERT protocol achieved a score of 8 on the PEDro scale. The safety protocol showed positive results. In this case, the introduction of early intensive out-of-bed mobilization accelerated the return on walking function and patient independence [35,36]. However, it is important to mention a more recent result of the protocol. Despite the positive results of the AVERT protocol on early mobilization of patients after hemorrhagic stroke, this recent study by Bernhardt et al., (2015) showed much worse results. In most patients, the first mobilization occurred within 24 h. The AVERT protocol of using a higher dose and very early mobilization was associated with a lower chance of a favorable and high outcome at 3 months [23]. This finding demonstrates the potential danger of early rehabilitation within the first 24 h after hemorrhage and is consistent with the aforementioned recent AHA guidelines.

There were several limitations to the systematic review that should be mentioned. First, many of the articles dealing with the rehabilitation of patients with ICH were largely concerned with the chronic condition. Second, in many cases, much valuable information about the timing of initiation of rehabilitation from admission to the hospital/stroke unit was missing in many cases. Many articles were noted, but had to be discarded at the initial stage due to the lack of much information, including mortality, type of brain hemorrhage or unclear information regarding rehabilitation and length of stay on the ward. Third, the control group was reported in only three cases. When designing future studies, attention should be paid to clarity of information and details. A description of the type of rehabilitation, its duration, the number of the exercise sessions and their type, the time at which the rehabilitation process started since the onset of the hemorrhage or the normalization of the condition can be valuable information showing the recovery process of patients with ICH [40].

The potentially small group size in half of the trials included in the review may have influenced the conclusions about the follow-up of patients after ICH. The results obtained may have been more favorable than if the study group had consisted of more than 1000 patients. This is a valid consideration for future studies with larger numbers of patients, as the outcomes of rehabilitation after ICH are poorly understood [41]. Mortality has been difficult to report in most studies. Many researchers do not report it or report it in a very limited way. A detailed description of mortality is important to determine the safety and validity of using early rehabilitation up to 24 and 48 h. Similar observations apply to the presence of control groups, which are not the rule in many studies of ICH and are of great importance in the context of the study’s results and conclusions. In the absence of a control group, no real conclusions can be drawn [42]. This is not a common pattern in medical research, but it may be noticeable in the field of hemorrhagic stroke and rehabilitation, where the number of patients, although increasing, is still small. Fulfilling the above criteria would have a better impact on clinical practice for healthcare teams working with patients after ICH in hospital wards.

We have identified gaps in the research presented in Table 1 above regarding the rehabilitation of patients after ICH. The largest relates to the duration of rehabilitation for patients. The lack of standardization of the rehabilitation protocol in the trial may mean that the duration of therapy varied from day to day or was not strictly followed and monitored. This poses a problem for clinical practice, as doctors, physiotherapists and occupational therapists may feel confused when prescribing rehabilitation. The remaining gaps relate to the detailed mortality rate mentioned above, the presence of a control group, or the lack of accurate reporting of the time after the onset of ICH, although the acute condition is reported. The final limitation of our review is that we did not include articles with closed access. This is due to the cost of obtaining full access to them and the frequent difficulty in finding them. In the future, another review on this topic, updating the current knowledge, should include both open and closed access articles, so that the results and the conclusions drawn are as true and in line with the real state as possible.

Based on the physical therapy methods and techniques presented, it is not entirely clear which type of rehabilitation and training protocol is most effective in restoring motor function in post-stroke patients. One interesting approach that has been shown to be increasingly effective is the use of focal muscle vibration (rMV). These vibrations have a stimulating effect on the processes of brain plasticity and long-term motor recovery. To date, research in this area has focused on chronic stroke, which has been positively evaluated and proven [43]. Referring to the functional and structural changes in neuronal networks that occur in the first hours after stroke onset, Toscano et al., (2019) conducted a study using rMV in patients in the acute phase. The study was conducted on ischemic and hemorrhagic stroke patients within 72 h of stroke onset. In addition to focal muscle vibration, patients were provided with rehabilitation. The results of the intervention (rMV) showed that it is a way to improve motor performance in patients with a recent stroke. According to the authors, this is due to direct effects on the ipsilesional motor cortex [44,45]. Results were favorable in post-stroke patients regardless of other baseline clinical status or various stroke characteristics. The combination of conventional rehabilitation and repetitive focal muscle vibration (rMV) can influence positive health outcomes in patients with recent strokes, including hemorrhagic stroke, and this therapy is safe for patients and easy to perform [45].

The results of our systematic review address a common concern among clinicians regarding the use of early rehabilitation in post-stroke patients [14]. The 2022 AHA guidelines on ICH have not changed much since the 2015 edition. The recommendations for rehabilitation tell us to implement rehabilitation quickly. This has implications for speeding up discharge from the hospital and receiving further therapy or rehabilitation recommendations at home to facilitate recovery. Attention should be paid to the timing of the start of rehabilitation to avoid wasting valuable time on brain recovery and plasticity. It is important to plan and implement neurorehabilitation 24–48 h after the hemorrhagic event. However, very intensive and vigorous rehabilitation should not be started within the first 24 h after a hemorrhagic stroke [6,46,47]. It is important to conduct more research on early rehabilitation in acute ICH patients, as more research is conducted on chronic patients [48,49,50].

## 5. Conclusions

Early rehabilitation after ICH can result in improved ADLs, motor function, functional independence, quality of life, gait improvement, improved trunk control and lower mortality. Late initiation of rehabilitation can result in permanent disability, increased mortality, and prolonged hospitalization. Lack of rehabilitation, or delayed rehabilitation at a very distant date, is more dangerous than early rehabilitation in patients with ICH. It is very important that rehabilitation be carried out carefully and with all safety precautions. The patient’s condition should be normalized and continuously monitored in order to start the early rehabilitation process. When the patient’s medical condition is relatively stable, active rehabilitation can begin. Intensive rehabilitation initiated in the acute phase, within 24–48 or even 72 h after the onset of ICH, accelerates the return to independence, gait and functional performance. It improves survival and functional outcomes 6 months after stroke. It improves survival and functional outcomes 6 months after stroke. There is a need for more detailed research on rehabilitation for acute ICH patients. The protocol for acute rehabilitation should be standardized, including the onset of rehabilitation and recommendations for specific therapies and methods.

## Figures and Tables

**Table 1:** A summary description of the studies included in the review.

	Author (Year) Country PEDro Scale	Type of Study	Number of Participant/Characteristics	Time of Reh since ICH/Duration of the Study	Rehabilitation Protocol /Duration	Mortality Rate	Methods	Main Benefits
**1**	Bai et al., 2011, China [5]. PEDro: 5/10	A prospective, randomized, single-blinded trial	*n* = 364 All patients with ICH ER/CG 181/183	10–11 days/ 6 months	three-stage rehabilitation program (physical therapy, occupational therapy, task-based ADL, basic exercise, learning to walk, balance)/45 min. for 5 days a week	ER: 2 CG: 5	- FMA - MBI	Early rehabilitation achieve better results in ADL, FMA and MBI scores and motor function
**2**	Yen et al., 2020, Taiwan [25]. PEDro: none	Prospective, assessor-blinded, randomized controlled trial	*n* = 60 Mild and moderate patients with ICH ER/CG	1–2 days/3 month	Early mobilization (EM), within 24–72 h of stroke onset And standard early rehabilitation (SER)/30 min., once a day, 5 days a week	0	- FIM - FAC - LOS - Postural Assessment Scale for Stroke Patients	ER improves early functional independence
**3**	Matsubara et al., 2021, Japan [18]. PEDro: none	Retrospective, Comparison studies	*n* = 3112 ICH/CI 1500/1612	Patients separated according to stroke onset: 7–13 days 14–20 days 21–27 days 28–34 days 35–41 days 42–48 days 49–55 days 56–50 days /8 weeks	Full-time integrated treatment (FIT) program at a comprehensive inpatient rehabilitation unit/ 2–3 h for 7 days a week	0	- FIM - LOS	More improvement in self-care was seen in ICH patients There is no difference in acute rehabilitation between the ICH group and the CI group
**4**	Sun et al., 2022, China [30]. PEDro: none	Not specified	*n* = 85,664 ICH in acute state	Acute state/over 15 days	Conventional functional rehabilitation in rehabilitation and stroke wards (acute hospitalization)/Lack of information	2015 (2.3%)	- GCS - LOS - mRS	ICH patients with hypertension: longer hospital stays and lower mortality figures
**5**	Epple et al., 2020, Germany [31]. PEDro: 7/10	Randomized controlled trial	*n* = 40 ICH/CI ER/CG 20/20 Acute state	72 h after stroke onset/ 9 days	Vojta therapy/40 min. per day, totally 7 sessions	3	- TCT - NIHSS - CBS - Measupes - mRS - BI	Improved postural control with Vojta therapy vs. standard physiotherapy
**6**	Wei et al., 2022 China [32]. PEDro: none	Randomised controlled trial	*n* = 100 ICH due to hypertension/CG	48 h after stabilization of health condition/lack of information	Shengxin acupuncture rehabilitation training/not standardized (several times a day, 6 cycles of acupuncture)	Lack of information	- MAS - FMA	better clinical results in neurological parameters, daily living and quality of life, and hypertension treatment
**7**	Xiong et al., 2021, China [33]. PEDro: none	Randomised controlled trial	*n* = 85 ICH ER/CG 41/44	48 h after stabilization of health condition/lack of information	Conventional rehabilitation (including massage, active and passive training and rehabilitation along with early hyperbaric oxygen therapy/**duration not standardized**	Lack of information	- NIHSS - BI - FMA - GOS - Cerebral blood flow	Hyperbaric oxygen therapy with rehabilitation training accelerates the recovery of neurological function in ICH with hypertension; improves cerebral blood flow and prognosis
**8**	Zheng et al., 2021, China [28]. PEDro: none	Randomised controlled trial	*n* = 112 ICH ER/CG 56/56	Acute state/ 2 weeks	Training of ADL, Intervention for limb activities, complex rehabilitation.	Lack of information	- NIHSS - FMA - HAMD - ADL - Care—patient satisfaction levels	Better neurological function, limb function and ADL in patients with ICH Alleviation of an adverse psychological mood
**9**	Ogata et al., 2015, Japan [34]. PEDro: none	Prospective study	*n* = 270 ICH ER/CG	Acute state Begin rehabilitation after 7 days/lack of information.	Rehabilitation with the robotic exoskeleton HAL.	156	- BS - BI - FIM	Improved functional recovery in patients with right-hemisphere ICH compared to left-hemisphere ICH
**10**	Bernhardt et al., 2008, Australia [35]. PEDro: 8/10	Prospective, open, randomized, controlled-trial, blinded-outcome assessment	*n* = 71 ICH/CI 9/62 ER/CG 33/38	Early mobilization within 24 h of symptom onset/14 days or until discharge	Conventional rehabilitation and the AVERT Protocol, /2 times a day 6 days a week	11	- NIHSS - mRS	The AVERT protocol, safe and feasible
**11**	Cumming et al., 2011, Australia [36]. PEDro: 8/10	Randomized controlled trial	*n* = 71 ICH/CI 9/62 ER/CG	24 h after ICH/CI /14 days or until discharge	Conventional rehabilitation and the AVERT Protocol, in which mobilization 24 h of the onset of symptoms/Lack of information	6	- NIHSS - mRS - BI - MAS - FIM - RMA	Faster return to independent walking and functional recovery
**12**	Liu et al., 2014, China [4]. PEDro: 8/10	Randomized controlled study, with blinded assessment of outcome	*n* = 326 ICH ER/CG	Within 48 h/lack of information	Conventional poststroke rehabilitation (rehabilitation ward) /16 times a month for 60 min per session.	Group with intervention VER—1 Group with standard care—12	- Survival rate - SF-36 - BI - Zung self-rated anxiety scale	Early rehabilitation improves survival and functional outcomes 6 months after ICH
**13**	Capo-Lupo et al., 2020, USA [27]. PEDro: none	Retrospective data analysis of prospectively collected data from an ongoing observational cohort study.	*n* = 203 ICH Acute state	Lack of information /lack of information	The average time to the start of acute rehabilitation (2 to 6 days after ICH) was 2 days/lack of information	Lack of information	- mRS - Glo. Di. S - NIHSS - GCS - The ICH score, and ventilator-free days	Earlier acute rehabilitation therapy (2 to 6 days) can reduce disability after ICH

Abbreviations: Scale: FMA—Fugl–Meyer Assessment; mRS- The Modified Rankin Scale; NIHSS—The National Institute of Health Stroke Scale; GCS—The Glasgow Coma Scale; BI-Barthel Index; SF-36—The 36-item Short Form Questionnaire; RMA—Rivermead Motor Assessment; FIM-Functional Independence Measure; MSAS-The mobility scale for acute stroke; BS—Brunnstrom Stages of Stroke Recovery; ADL—Activities of daily living; HAM-D-Hamilton Depression Rating Scale; GOS-Glasgow Outcome Scale; MAS—The Modified Ashworth Scale; CBS—Catherine Bergego Scale; MESUPES—Motor Evaluation Scale for Upper Extremity in Stroke Patients; TCT—Trunk Control Test; EuroQol EQ-5D-3L- European Quality of Life; LOS—Length of stay at the facility; FAC—Functional Ambulation Category; MBI—Modified Barthel Index; Glo. Di. S—Global disability with scores; Group: CT-Control Group, ER—Early Rehabilitation.

**Table 2:** Details of manual search in medical databases.

Database	Search Terms	Total Records Recovered
PubMed	Early rehabilitation of intracerebral hemorrhage (ICH) Acute rehabilitation of intracerebral hemorrhage (ICH) Rehabilitation of intracerebral hemorrhage (ICH) Mortality of rehabilitation intracerebral hemorrhage (ICH)	331
PubMedCentral		571
Medline		122
Cochrane Library		163
Embase		31
Scopus		59

**Table 3:** Search results of a qualified systematic review of studies on rehabilitation and ICH.

Database	Article Qualified for Review
PubMed	4
PubMedCentral	5
Medline	3
Cochrane Library	1
Embase	0
Scopus	0
